# Dopamine D1/D2 receptors do not mediate the expression of conditioned place preference induced by the aftereffect of wheel running

**DOI:** 10.1186/s12868-014-0124-4

**Published:** 2014-11-19

**Authors:** Alexandra Trost, Wolfgang Hauber

**Affiliations:** Department Animal Physiology, University of Stuttgart, Pfaffenwaldring 57, D-70550 Stuttgart, Germany

**Keywords:** Wheel running, Aftereffect, Reinforcement, Dopamine, Rat

## Abstract

**Background:**

Rats lever-press for access to running wheels suggesting that wheel running by itself is reinforcing. Furthermore, pairings of an episode of wheel running and subsequent confinement in a specific environment can establish a conditioned place preference (CPP). This finding implies that the reinforcing effects of wheel running outlast the actual occurrence of physical activity, a phenomenon referred to as aftereffect of wheel running. Aftereffect-induced CPP involves Pavlovian conditioning, i.e. repeated pairings of the aftereffect of wheel running with a specific environment creates a learned association between aftereffect and environment and, in turn, a preference for that environment. Given the involvement of dopamine systems in mediating effects of Pavlovian stimuli on appetitive behavior, a role of dopamine in mediating aftereffect-induced CPP seems plausible. Here we assessed whether the mixed D1/D2 receptor antagonist flupenthixol (0.25 mg/kg, i.p.) can block the expression of an aftereffect-induced CPP.

**Results:**

In line with earlier studies, our results demonstrate that rats displayed a conditioned preference for environments paired with the aftereffect of wheel running and further show that the magnitude of CPP was not related to the wheel running rate. Furthermore, we found that flupenthixol (0.25 mg/kg, i.p.) reduced locomotor activity but did not attenuate the expression of an aftereffect-induced CPP.

**Conclusion:**

The expression of a CPP produced by the aftereffect of wheel running seems not to depend on dopamine D1/D2 receptor activation.

## Background

Considerable evidence suggests that wheel running in rodents has reinforcing effects. For instance, rats lever-press for access to running wheels, i.e., the opportunity to run acts as a reinforcer that maintains operant responding [1-3]. Interestingly, those rats that developed the highest rates of running during wheel access also maintained the most stable and highest rates of lever pressing [3]. Furthermore, unrestricted wheel running can produce compulsive patterns of responding suggesting that wheel running has parallels with drug addiction [4]. Consistent with this notion, wheel running can act as a hedonic substitution for other reinforcers and attenuate self-administration of cocaine [5]. Considerable evidence suggests that opioid and dopamine systems are involved in mediating reinforcing effects of wheel running [6]. For instance, brief periods of wheel running increased striatal dopamine metabolism [7] and prior experience with wheel running produced cross-tolerance to the rewarding effects of morphine [8].

Remarkably, wheel running not only reinforces the behavior that generates it but also produces a preference for the environment that follows it [2]. For instance, pairings of an episode of wheel running and subsequent confinement in a specific environment can establish a conditioned place preference (CPP). These findings imply that the reinforcing effects of wheel running outlast the actual occurrence of physical activity, a phenomenon referred to as aftereffect of wheel running [2,8-10]. As with reinforcing effects of wheel running by itself, reinforcing effects of the aftereffect of wheel running are supported by opioid systems. For instance, naloxone, a μ-opioid receptor antagonist, attenuated the acquisition and expression of an aftereffect-induced CPP [9].

CPP involves Pavlovian conditioning, i.e., repeated pairings of the aftereffect induced by wheel running with a specific environment creates a learned association between aftereffect and environment and, in turn, a preference for that environment [11]. It is well known that dopamine systems play a critical role in mediating effects of Pavlovian stimuli on appetitive behavior [12], e.g., dopamine receptor blockade attenuated conditioned approach behavior [13]. Furthermore, through activation of dopamine systems, Pavlovian stimuli can both amplify and direct instrumental responding, phenomena termed as general and outcome-selective Pavlovian-instrumental transfer [14-18]. However, the role of dopamine in aftereffect-induced CPP is unknown at present. Here we investigated whether a blockade of dopamine receptors by systemic administration of the mixed D1/D2 receptor antagonist flupenthixol can block the expression of a CPP established by pairings of the wheel running aftereffect with a specific environment. Given the crucial role of dopaminergic systems in mediating effects of Pavlovian stimuli on appetitive behavior, flupenthixol should attenuate the expression of an aftereffect-induced CPP.

Of note, monoamine systems have been implicated in exercise addiction in humans [19]. Environmental stimuli play a critical role in maintaining addictive behavior and the CPP paradigm provides an important tool to analyze behavioral effects of contextual stimuli associated with drug cues [11]. Therefore, the present study could provide clues as to whether dopamine systems mediate behavioral effects of contextual stimuli associated with the aftereffect of physical activity.

## Results

### Conditioned place preference

**Figure 1:**
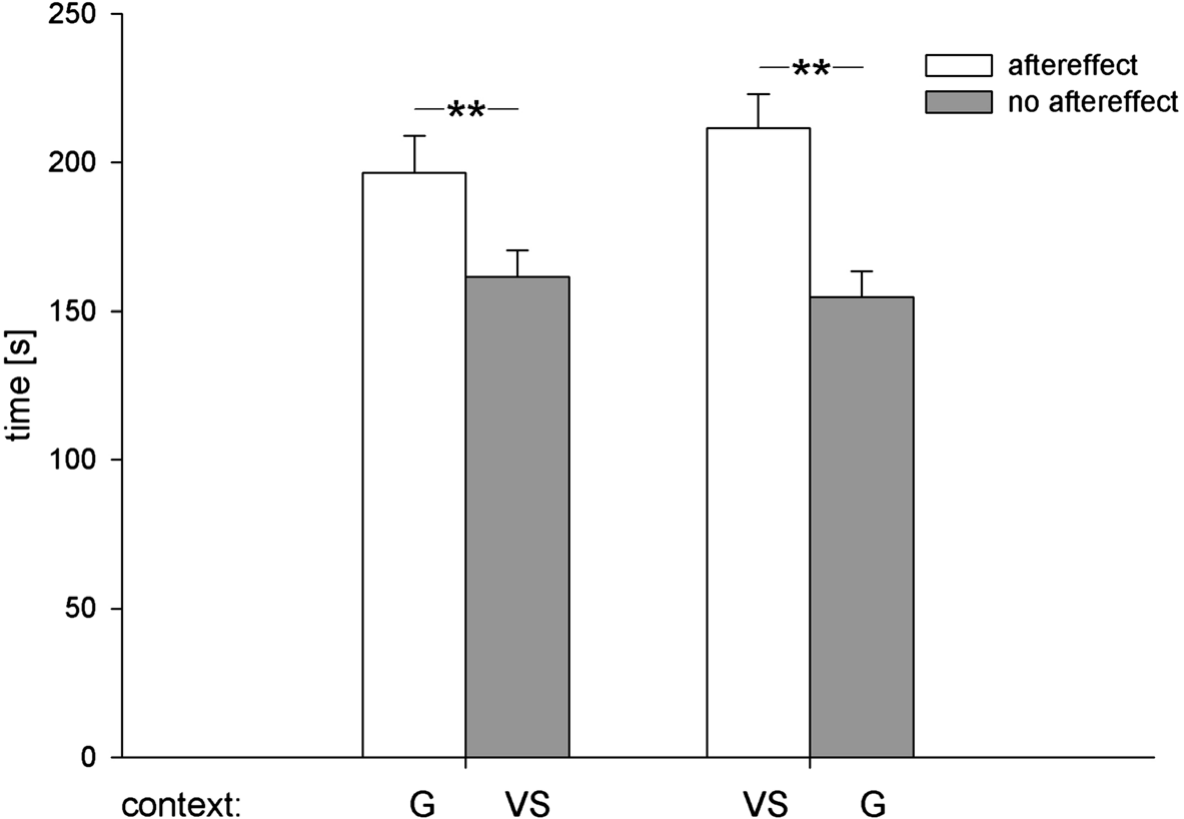
**Aftereffect-induced CPP.** Mean times (± SEM) rats spent in the chamber paired with the aftereffect of 2 h wheel running vs. the control chamber as a function of the chamber context (N = 32; G: grey; VS: vertical stripes). An ANOVA revealed a significant effect of aftereffect (**p < 0.0005), but no effect of chamber context and no aftereffect × chamber context interaction.

In the place preference test, rats preferred the chamber paired with the aftereffect of wheel running over the control chamber. Furthermore, the context identity did not influence place preference (Figure 1). A statistical analysis confirmed this description. A two-way ANOVA with aftereffect and context identity as factors revealed a significant effect of aftereffect (F_(1,30)_ =16.18; p < 0.0005), no effect of context identity (F_(1,30)_ =0.2; n.s.) and no aftereffect × context identity interaction (F_(1,30)_ =0.9; n.s.). Furthermore, CPP magnitude did not correlate with wheel running rate. Individual distances covered during wheel running on the final training day and time spent in the chamber paired with the aftereffect did not correlate (Pearson’s *r* = −0.25; n.s).

### Flupenthixol effects on conditioned place preference

**Figure 2:**
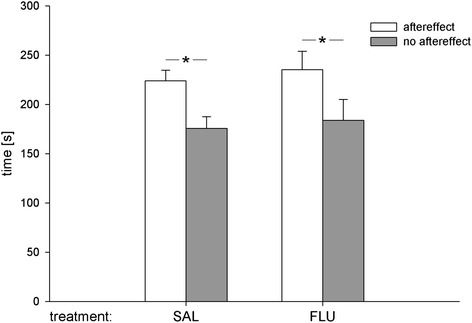
**Effects of flupenthixol on aftereffect-induced CPP.** Mean times (± SEM) spent in the chamber paired with the aftereffect of 2 h wheel running vs. the control chamber in animals that received saline (1 ml/kg, i.p., n = 16) or flupenthixol (0.25 mg/kg, i.p., n = 16) 60 min prior CPP testing. An ANOVA revealed a significant effect of aftereffect (*p < 0.05), but no effect of treatment and no aftereffect × treatment interaction.

Results further demonstrate that, like saline controls, rats pretreated with flupenthixol displayed a preference for the chamber experienced under the aftereffect of wheel running over the chamber experienced without prior wheel running (Figure 2). A statistical analysis confirmed this description. An ANOVA with aftereffect and treatment as factors revealed a significant effect of aftereffect (F_(1,30)_ =6.44; p < 0.05), but not of treatment (F_(1,30)_ =0.67; n.s.) and no aftereffect × treatment interaction (F_(1,30)_ =0.006; n.s.).

### Flupenthixol effects on locomotor activity

**Figure 3:**
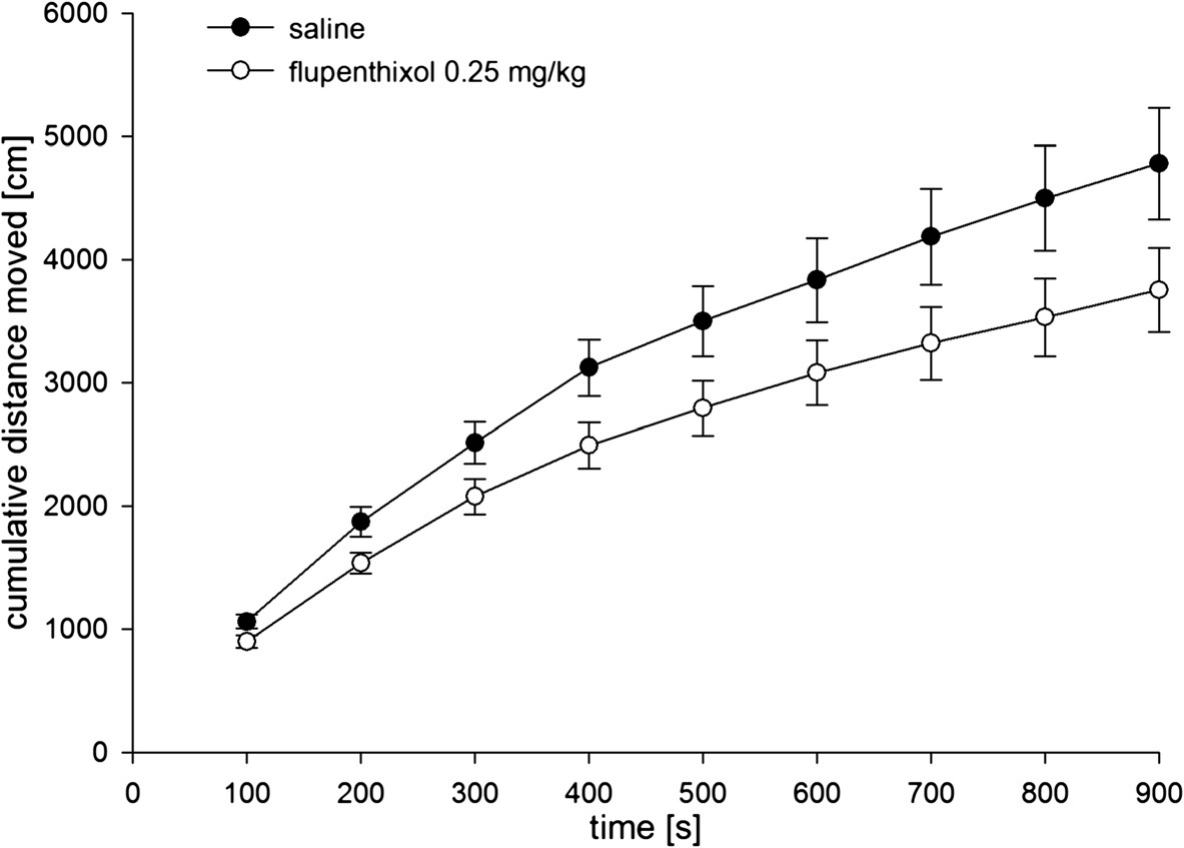
**Effects of flupenthixol on locomotor activity.** Mean cumulative distance (± SEM) moved in an open field in animals (n = 12) under saline (1 ml/kg, i.p.) vs. flupenthixol (0.25 mg/kg, i.p.) administered 60 min prior CPP testing. An ANOVA revealed a trend for an effect of treatment (F_(1,11)_ =4.69; p =0.053) and a significant treatment × time interaction (F_(8,88)_ =2.06; p < 0.05).

In a separate group of rats, we found that the dose of flupenthixol used in the CPP experiment reduced locomotor activity in an open field (Figure 3). Accordingly, an ANOVA on cumulated distance moved with treatment and time as factors revealed an almost significant effect of treatment (F_(1,11)_ =4.69; p =0.053) and a significant treatment × time interaction (F_(8,88)_ =2.06; p < 0.05).

## Discussion

Our results demonstrate that rats displayed a conditioned preference for environments paired with the aftereffect of wheel running and further show that the CPP magnitude was not related to the wheel running rate. Moreover, we found that the mixed dopamine D1/D2 receptor antagonist flupenthixol did not block the expression of CPP produced by the aftereffect of wheel running.

Consistent with previous studies using a similar protocol [2,8-10], the aftereffect generated by 2 h wheel running induced a CPP. The CPP effect size in our study was strong (*r* =0.59). Of note, earlier studies [2,8-10] used vertical and horizontal stripe chambers for CPP. Because rats tend to prefer vertical over horizontal stripe chambers [20], CPP may be influenced by context configuration even if the experimental design controlled for such a bias [2]. In our pilot studies, rats did not show spontaneous chamber preferences (monochrome grey vs. vertical black lines) suggesting that context configuration was balanced in the CPP apparatus used here. Importantly, data from the experiments reported here provided no evidence for an influence of context identity on aftereffect-induced CPP, i.e. statistical analysis demonstrated that the chamber context *per se* had no effect on CPP and did not interact with the aftereffect. Yet, due to considerable methodological differences in the experimental design (within vs. between subjects) and the place preference test system (2 vs. 3 chamber design), it is difficult to assess whether our balanced context configuration accounts for the, relative to previous studies [2,8-10], marked CPP effect size. Furthermore, as reported in other studies using 3-chamber CPP apparatus [21,22], animals spent some time in center compartment connecting the chambers. Nevertheless, reinforcing effects of a given stimulus such amphetamine can be reliably assessed in a CPP apparatus with either a 2- or 3-chamber design [23,24]. However, we cannot rule out that the use of a 3-chamber CPP apparatus influenced CPP effect size to some extent.

Our observation that the aftereffect of 2 h wheel running generated a robust CPP adds further support to the notion that the aftereffect is reinforcing as detected by a number of studies using various wheel running paradigms [2,10,25,26]. Remarkably, we found no evidence for a relationship between CPP magnitude and wheel running rate within 2 h. In line with this observation, higher rates of wheel running did not correlate with stronger CPP [2]. However, the relationship between CPP and reinforcing efficacy of stimuli bears a number of complexities. For instance, the magnitude of CPP induced by drug reinforcement in a given rat seems not to reflect the magnitude of its reinforcing efficacy measured by drug self-administration in the same rat [11]. Furthermore, while there is a large body of evidence suggesting that physical activity is a natural reinforcer in rodents [6], it is not clear whether there exist simple, e.g. linear, dose–response relationships between the intensity or duration of physical activity such as wheel running and its reinforcing efficacy and the resulting aftereffect. Studies in humans on associations between activity doses and affective responses also point to complex relationships between aerobic exercise and positive or negative affect that do not reflect simple inverted U-shaped curves [27,28].

We further demonstrated that the mixed dopamine D1/D2 receptor antagonist flupenthixol did not block the expression of an aftereffect-induced CPP. In addition, results show that the dose of flupenthixol used here (0.25 mg/kg) was behaviorally effective, i.e., reduced locomotor activity. In line with this notion, flupenthixol at 0.3 mg/kg but not at 0.15 mg/kg produced a within-session decline of locomotor activity [29]. Likewise, flupenthixol at 0.2 and 0.3 mg/kg was able to reduce the expression of CPP induced by testosterone [30] suggesting that under-dosing of flupenthixol seems unlikely to account for its failure to attenuate aftereffect-induced CPP. By contrast, our pilot experiments suggest that flupenthixol at doses of 0.4 mg/kg and higher may be not appropriate as they cause pronounced locomotor inhibition that interferes with CPP.

In the CPP paradigm used here, reinforcing aftereffects of a natural reinforcer such as wheel running was paired with exposure to specific environmental stimuli and this stimulus came to elicit a conditioned approach in the place preference test. The ability of a stimulus previously paired with reinforcement to acquire incentive motivational properties is mediated by Pavlovian learning and plays a key role not only in CPP but also in related phenomena such as Pavlovian-instrumental transfer [12]. The ventral striatum is modulated by dopamine and opioid systems [31] and represents a key element of the neural circuit that mediates expression of a CPP induced by natural reinforcers [32]. There is considerable evidence for a dopaminergic involvement in governing the effects of Pavlovian stimuli on appetitive behavior, e.g., dopamine receptor activation enhanced while dopamine receptor blockade attenuated conditioned approach behavior [13,33]. Furthermore, a number of studies point to a role of dopamine both in acquisition and expression of CPP generated by drug reinforcers. For instance, the acquisition of amphetamine-induced CPP requires both D1 and D2 receptor activation, while the expression of amphetamine-induced CPP selectively depends on D1 receptor activation [34]. However, a substantial number of studies suggests that dopamine depletion or dopamine receptor blockade failed to block CPP induced by cocaine, methylphenidate or nomifensine [35-39] pointing to an involvement of non-dopaminergic mechanisms in mediating reinforcing effects of dopaminergic drugs measured in CPP. As yet only a few studies examined the role of dopamine in the acquisition of CPP established by natural reinforcers. Results are mixed, e.g., dopamine D1/D2 receptor antagonists blocked the acquisition of food-reinforced CPP [40] but not CPP induced by paced mating behavior [41], while specific D2 receptor antagonists even facilitated the acquisition of food-reinforced CPP [42]. By contrast, the effects of dopamine receptor ligands on the acquisition of aftereffect-induced CPP are as yet unknown. Our results are, to our knowledge, the first to show that the expression of CPP induced by the aftereffect, i.e. a prominent natural reinforcer, does not depend on dopamine D1/D2 receptor activation. Further studies are required to assess whether or not the expression of CPP induced by other natural reinforcers than the aftereffect requires dopamine D1/D2 receptor activity. Moreover, it is possible that opioid rather than dopamine receptor activity supports the expression of aftereffect-induced CPP. It is well known that, by mediating the hedonic response to natural reinforcers, opioid receptor activity governs the acquisition of CPP [43]. For instance, opioid receptor antagonists attenuated the acquisition of a CPP induced by water [44] and the aftereffect of wheel running [9]. Furthermore, it has been speculated that the expression of CPP may also rely on opioid receptor activity, i.e., via an opioid receptor-mediated evocation of a hedonic response that elicits approach to the associated chamber [43]. Furthermore, recent data suggest that μ-opioid receptor-mediated signals not only govern hedonic responses but are also involved in mediating the incentive effects of Pavlovian stimuli on appetitive behavior [45]. Collectively, these findings point to the possibility that, by mediating both incentive and hedonic effects of Pavlovian stimuli, opioid receptor activation could support the expression of an aftereffect-induced CPP.

## Conclusions

The aftereffect of wheel running for 2 h is reinforcing and can establish a CPP, however, expression of an aftereffect-induced CPP seems not to depend on dopamine D1/D2 receptor activation. Monoamine systems have been implicated in exercise addiction in humans [19]. As environmental stimuli play a critical role in maintaining addictive behavior the CPP paradigm provides an important tool to analyze behavioral effects of contextual stimuli associated with drug cues [11]. Our results suggest that dopamine D1/D2 receptor activity may not maintain exercise addiction in humans by mediating the behavioral effects of contextual stimuli associated with the aftereffect of physical activity.

## Methods

All animal experiments were conducted according to the German Law on Animal Protection and approved by the local council of animal care (Regierungspraesidium Stuttgart).

### Subjects

Male Sprague–Dawley rats (Janvier, Le Genest St. Isle, France, N = 32) weighing 271.6 ± 5.5 g at the beginning of the experiments were used for CPP experiments. They were housed in pairs in transparent cages (39 × 55 × 27 cm; Ebeco, Castrop-Rauxel, Germany) with standard metal lids. During a limited period of 4 weeks prior to the onset of behavioral experiments, cages were supplied with custom-made lids with an integrated running wheel (diameter: 31.5 cm, width: 10 cm). Rats had *ad libitum* access to water; food (standard maintenance chow; Altromin, Lage, Germany) was restricted to 15 g per animal and day. Temperature (21 **±** 2°C) and humidity (45-50%) were kept constant in the animal house. A 14:10-h light–dark cycle (lights on at 8.00 a.m.) was used; behavioral experiments were performed during light period of the light–dark cycle.

A separate group of male Sprague–Dawley rats (Janvier, Le Genest St. Isle, France, n = 12) weighing 272.9 ± 2.8 g at the beginning of the experiment was used for a control experiment to assess the effects of flupenthixol on locomotor activity. They were housed in groups of four in transparent cages (39 × 55 × 27 cm; Ebeco, Castrop-Rauxel, Germany) with standard metal lids as described above.

### Apparatus

#### Running wheels

Running wheels were used during conditioning enclosed by a transparent plastic box to prevent rats from leaving the running wheels. Running wheels (diameter 31.5 cm; 10 cm width) were identical to those used for habituation in the home cage.

#### Place preference conditioning apparatus

A 3-chamber place preference system was employed (TSE, Bad Homburg, Germany) which consisted of a “vertical” and “grey” chamber (each 30 cm long × 25 cm wide × 32 cm deep) connected by a center compartment (11 cm long × 25 cm wide × 32 cm deep). The vertical and grey chambers as well as the center compartment were distinct in wall patterns and floor textures. The vertical chamber included walls with vertical black and white stripes (2 cm width) and a metal floor with square holes (1 cm^2^, 2 cm apart), the grey chamber included grey walls and a metal floor with circular holes (diameter 0.5 cm, 5 cm apart), the center compartment had white walls and an unperforated metal floor. The lid of each chamber and of the center compartment consisted of a plastic panel. During place preference conditioning, access to the center compartment was blocked to confine a rat to a particular chamber.

#### Open field

Locomotor activity was recorded in an open field (68 × 68 cm). The testing area was illuminated by red light and surrounded by a cubicle providing optical and acoustical isolation. Locomotor activity was monitored by a video recording system (EthoVision XT 8.5, Noldus, Wageningen, Netherlands) and analyzed off-line.

### Procedure

#### Place preference conditioning

Prior to the onset of place preference conditioning, rats were adapted in their home cages to the running wheels. During place preference conditioning, each rat received 6 pairings of one of both chambers (vertical or grey context) and the aftereffect of wheel running and 6 unpaired exposures to the other chamber with the different context. One pairing was given per day with paired and unpaired trials alternated. Chamber context/aftereffect pairings were counterbalanced across animals. During a paired trial, an animal was confined for 2 h in a running wheel integrated in a box and immediately thereafter placed into the respective chamber, during an unpaired trial, an animal was placed for 2 h in a small cage (42.5 cm × 26.5 cm × 25 cm) before being transferred into the respective chamber. Covered distances in running wheels were recorded from all animals on days 4–6 (N = 32), from a subgroup (n = 16) on days 1–3.

#### Place preference test

One day after completion of place preference conditioning, each animal was given a place preference test for 10 min with free access to both chambers. At the beginning of the place preference test, an animal was placed into the middle of the center compartment with the body position oriented to compartment wall. A chamber visit was counted if all 4 paws were within a particular chamber.

#### Place preference test under drug

Three days after the first place preference test, a second place preference test was performed as described above but with prior drug administration, i.e. animals received injections of either flupenthixol (0.25 mg/kg, n = 16) or saline (1 ml/kg, n = 16). Animals were assigned to treatment groups in a pseudo-random manner counterbalanced to chamber context.

#### Open field

Effects of flupethixol (0.25 mg/kg, i.p.) and vehicle (1 ml/kg, i.p.) on locomotor activity were tested 60 min after administration using a within-subjects design. The day before testing each animal was placed individually into the open field for habituation (10 min). On the subsequent test day 1, and 4 days later, on test day 2, locomotor activity was recorded in a 15 min session, respectively. On test days 1 and 2, each rat received counterbalanced administration of vehicle and flupenthixol. Cumulated distance moved in 100 s-bins is given.

#### Drugs

Flupenthixol (cis-(Z)-Flupenthixoldihydrochlorid, Sigma Aldrich) was dissolved in physiological saline (Braun, Melsungen Germany). Intraperitoneal injections were in a volume of 1 ml/kg 60 min. prior test onset.

### Statistical analysis

Distance covered by each animal in the wheel running box within 2 h was recorded as well as the time spent in each chamber during the place preference test and the cumulative distance moved in the open field. Means ± standard error of the mean (SEM) are given. Data were subjected a repeated-measures analysis of variance (ANOVA). Based on a planned comparison using the t-statistic [46], the effect size *r* of the aftereffect on time spent in the paired vs. unpaired chamber was calculated according to the following equation: r = √t^2^/t^2^ + d*f* [47]. Statistical significance was assessed against a type I error rate of 0.05. All statistical computations were carried out with STATISTICA 7.1 (StatSoft, Inc., Tulsa, USA).

## Abbreviation

CPP: Conditioned place preference
